# Evaluating data partitioning strategies for accurate prediction of protein-ligand binding free energy changes in mutated proteins

**DOI:** 10.1016/j.csbj.2025.10.020

**Published:** 2025-10-14

**Authors:** Liangxu Xie, Guoming Bao, Dawei Zhang, Lei Xu, Xiaojun Xu, Shan Chang

**Affiliations:** Institute of Bioinformatics and Medical Engineering, Jiangsu University of Technology, Changzhou 213001, China

**Keywords:** Mutant proteins, Relative free energy, Protein language model, Data partitioning strategy

## Abstract

Accurate prediction of the relative free energy of protein-ligand binding, especially regarding protein mutations, is vital for drug design and interpreting drug resistance. However, machine learning (ML) / deep learning (DL) methods often struggle with generalization due to dataset partitioning strategy. Random data partitioning potentially produces spuriously high correlations that inflate performance estimates. UniProt-based splitting preserves data independence but lacks high prediction accuracy. In this study, we first evaluate six distinct ML/DL models on the MdrDB database using two data partitioning methods. Protein sequences are embedded using the ESM-2 protein large language model, integrating wild-type and mutant features. Although all models show high predictive correlations (Pearson coefficients up to 0.70) under random partitioning, their performance declines with UniProt-based partitioning. To address this issue, we propose a query-anchor pairwise learning framework, utilizing known states as anchor points for predicting unknown query states. The proposed method is validated across three systems, revealing that even a small amount of reference data can significantly enhance prediction accuracy. This enhancement suggests that leveraging known states as anchor points allows for more precise predicting of unknown query states.

## Highlights

• Evaluated impact of different data partitioning strategies on predicting mutation-induced changes in binding free energy.
• UniProt-based partitioning reduces model prediction accuracy, highlighting potential overestimation from conventional methods.
• Proposed an anchor-query partitioning framework, leveraging limited reference data to improve predictive generalization.

## Graphical Abstract

**Figure:**
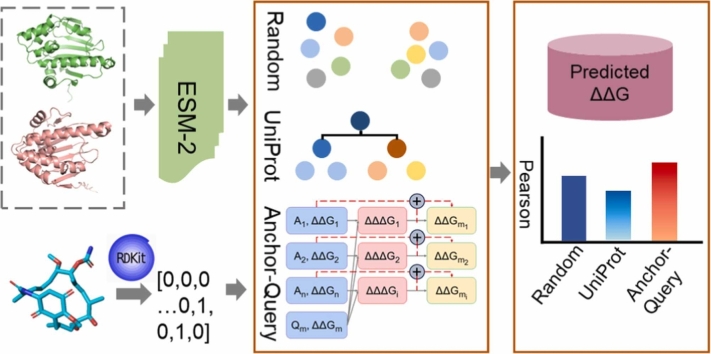

## 1 Introduction

Protein-ligand binding affinity lies at the heart of biomolecular recognition, regulation, and the mechanisms underlying drug action. Accurately predicting the relative free energy change (ΔΔG) caused by protein mutations not only helps to gain a deeper understanding of protein function and allosteric regulation mechanisms but also provides a key support for rational drug design, enzyme engineering, and interpretation of clinical gene variants [1], [2], [3], [4], [5]. With the continuous expansion of sequence and structure databases, deep learning (DL) methods have become an indispensable tool in molecular biophysics.

Computational methods have been widely used in the prediction of protein-ligand binding affinity [6], [7], [8], [9]. Predicting how point mutations affect protein-ligand binding affinity is a critical challenge in computational biology with direct applications in drug design and protein engineering. Molecular dynamics (MD) simulations with free energy perturbation (FEP) are considered the gold standard for computing free energy changes [10], [11]. However, FEP is computationally extensive and is sensitive to force field parameters and sampling adequacy [12]. A more efficient alternative, end-point binding free energy computation methods, such as MM/GBSA and MM/PBSA, provide a faster estimate of binding affinity. For instance, Sun et al. obtained reasonable accuracy in predicting the mutation effects of protein-ligand interactions [13]. Although these methods have some physical explanations and theoretical bases, they are mostly limited by high computational cost and sensitivity to the initial conformation in large-scale mutation scanning or virtual screening, which makes them difficult to meet the needs of high-throughput research. With the rapid development of artificial intelligence, machine learning (ML) and DL methods have greatly promoted progress in the field of molecular property prediction [14], [15]. ML algorithms such as the random forest [16] can effectively capture the nonlinear relationship between molecular features and experimental phenotypes. Various neural network structures have been widely used due to their ability to learn hierarchical features in complex high-dimensional data, and show robust performance in various bioinformatics tasks such as affinity prediction [17], [18], [19], [20], [21]. Machine learning (ML) based methods leverage biophysical data to predict the binding affinity with high efficiency [22], [23], [24]. A primary limitation of these approaches is data scarcity and poor generalization to unseen targets. The lack of large, high-quality structural data restricts model training, ultimately compromising predictive accuracy. Advances in deep learning for generating accurate 3D structures provide structural data sources for training models on mutation-induced free energy changes [25].

Significant progress in the field of protein structure prediction has advanced related research work. AlphaFold2 achieved high-precision prediction of protein tertiary structure from sequence to structure [26], [27]. The new version AlphaFold3 is expanded to the structure prediction of multi-chain complexes and protein-ligand complexes [28]. Meanwhile, protein language models have provided a new paradigm for protein representation learning [29]. The Evolutionary Scale Modeling (ESM) series employ large-scale Transformer architectures trained on massive protein sequence databases [30]. ESM achieves deep protein sequence embeddings and provides an improved expressiveness for protein sequences [31], [32], [33], [34]. ProtT5, based on a T5-style encoder-decoder framework, leveraged transfer learning from natural language processing to protein science. Trained on billions of sequences, ProtT5 demonstrated high expressive power in residue-level annotation, secondary structure prediction, and downstream applications, including protein-ligand binding affinity modeling [35], [36]. These protein language models enabled the generation of deep contextual embeddings of protein sequences [37], thereby enhancing protein characterization and supporting diverse downstream tasks such as structure prediction, binding interface, protein thermostability [38], and mutational effect analysis [39]. These models greatly promoted the development of the field of structure prediction and provided a new methodological foundation for downstream applications such as protein-ligand binding affinity prediction.

The computational prediction of binding affinity is a critical task in biophysics, with major applications in predicting both protein-ligand [40] and protein-protein interactions [41]. While significant progress has been made in predicting absolute protein-ligand binding affinity (ΔG), achieving sufficient accuracy for relative binding affinity (ΔΔG) remains a key challenge [42]. We focus exclusively on mutation-induced binding free energy changes (ΔΔG), defined as the difference in binding free energy between mutant and wild-type proteins. While success has been achieved in the field of absolute protein-ligand binding affinity prediction [43], the relative binding affinity [44], [45] still faces the challenge of sufficient accuracy. For binding affinity prediction, existing methods can be categorized into structure-based and sequence-based approaches [46]. Structure-based methods rely on three-dimensional structural data. For protein-protein interaction prediction, structure-based approaches integrate three-dimensional conformational features with docking scores [47]. DeepDDG employs a neural network trained on pre-defined molecular descriptors to predict the stability changes of proteins caused by point mutations [48]. Similarly, for protein-ligand affinity prediction, these methods analyze features derived from the spatial complex, such as docking poses and intermolecular contacts. The spatial structural information of protein-ligand complexes is also used for protein-ligand binding affinity prediction [49], [50]. Previous study reported that a machine learning model trained on structural features from molecular dynamics simulation achieves the best performance using CNN-LSTM with an accuracy of 0.841 [51]. However, due to the difficulties in obtaining experimental protein structure data, some scholars have turned to sequence-based methods [52]. Sequence-based methods have emerged as a powerful alternative, particularly to overcome the limitation of scarce experimental protein structures [53]. These approaches leverage amino acid sequences alone. For example, protein language models are used to extract embeddings for predicting protein-protein interactions [54], [55]. Likewise, sequence-based techniques are also being applied to the challenge of protein-ligand binding affinity prediction [56].

Our work specifically addresses mutation-induced changes in protein-ligand affinity (ΔΔG), which are directly relevant for studying drug resistance and mutational impacts on ligand binding. The impact of mutants on protein structure and drug discovery has already been realized [57]. Many efforts have been made in the field of structural biology to build sophisticated models of the impacts of mutations on protein-molecule interactions from a structural viewpoint [58], [59]. However, AlphaFold2 is limited in predicting the mutant structures of proteins [60]. They have delved deep into the three-dimensional structures of proteins and small molecules, analyzing the intricate details of their binding sites and the forces that govern their interactions [61], [62]. Computational algorithms have also been developed to simulate the effects of mutations on these interactions, taking into account factors like changes in electrostatic forces, steric hindrance, and conformational flexibility [63], [64]. Nevertheless, despite these extensive endeavors, these attempts have achieved only limited success or have restricted applicability. To address the limitations on crystal structures, strategies for encoding the differences between wild-type and mutant protein features are adopted [65]. Xin et al. proposed sequence-based neural network deep learning models to overcome the limitations of traditional methods that depend on protein crystal structures [66].

Previous studies reported very optimistic results, but the impact of dataset partition on the results has not been fully revealed. The existing evaluation system generally adopts a random data partitioning strategy, which may cause model performance to be overestimated and reduce the prediction accuracy when facing new target proteins. There are two key issues when predicting relative binding affinity. First, the impact of data partitioning schemes on model accuracy and generalization performance has not been systematically exploited. Second, in the field of mutation-dependent protein-ligand interaction prediction, there remains a notable absence of standardized methodologies for objective performance evaluation. Specifically, the field lacks consensus on how to properly utilize available experimental data and quantify confidence intervals when making predictions across different known datasets. In this study, we systematically investigated the impact of data partitioning strategies on the potential accuracy of predicting relative free energy changes of protein-ligand complexes. Utilizing the MdrDB database, which integrates extensive structural and experimental binding data for wild-type and mutant protein–ligand complexes, we extracted protein features using the ESM-2 model and encoded ligands with ECFP molecular fingerprints. The difference between wild-type and mutant features was concatenated with the ligand fingerprint as input. Six typical ML/DL methods were used: RF [58], SVR [67], GRU [68], BiLSTM [69], DNN [70], and Transformer encoder [71]. To comprehensively examine the impact of data partitioning strategies, we adopted two partitioning schemes: random partitioning and UniProt-based partitioning. In terms of model evaluation, we systematically compared the generalization performance of various models, aiming to answer a core scientific question: what accuracy potential can current mainstream ML/DL methods achieve in predicting protein-ligand binding free energy when faced with true distribution drift and data constraints? To enhance prediction performance in mutation-related binding affinity tasks, we propose a UniProt-based anchor-query partitioning strategy. This approach designates reference data as the anchors for the unknown states in the same UniProt group, while unknown unannotated states are treated as query data. By constructing pairwise inputs combining anchors and queries, our method enables more accurate prediction of relative binding affinities for novel mutations. Our partitioning strategy plays a critical role in protein-ligand binding prediction, particularly when reference data are available, offering measurable improvements in relative binding affinity estimation.

Our method is distinguished from existing structure-based predictors. mCSM-lig [72], which predicts binding affinity changes, requires 3D protein structures to calculate pre-defined molecular descriptors and graph-based signatures. In contrast, our approach is purely sequence-based, leveraging protein language model embeddings and a novel query-anchor framework to predict binding energy changes, eliminating the need for structural input or feature engineering. Our results highlight the importance of data partitioning in ML/DL-driven binding affinity studies, bridging gaps between known and unknown protein functional states.

## 2 Materials and methods

### 2.1 Dataset

The raw dataset utilized in this study is the MdrDB (Mutation-Dependent Response Database), which integrates information related to protein mutations and changes in small molecule binding affinity (ΔΔG) from multiple public sources, covering the structures and experimental data of various protein-ligand mutant complexes. After data cleaning, the dataset used contains 4179 protein-ligand mutant records, including wild-type protein sequences, mutant sequences, ligand molecules, and the corresponding experimentally measured binding free energy change (ΔΔG). The MdrDB dataset thus provides a robust foundation for evaluating modeling accuracy under different partitioning strategies in the present study. The MdrDB dataset used in this study comprises 4179 protein-ligand mutant records across multiple UniProt IDs. The experimentally reported relative binding free energy changes (ΔΔG) span a broad range, typically from approximately −4.5 kcal/mol (stabilizing mutations) to + 5.2 kcal/mol (destabilizing mutations), which encompasses both affinity-enhancing and affinity-reducing effects. However, high-confident structures of proteins for both wild and mutants may not exist at the same time. MdrDB also includes the mutant structures generated by PyMOL and AlphaFold 2.0. Therefore, we adopt a sequence-based method to avoid the use of 3D structures.

**Fig. 1:**
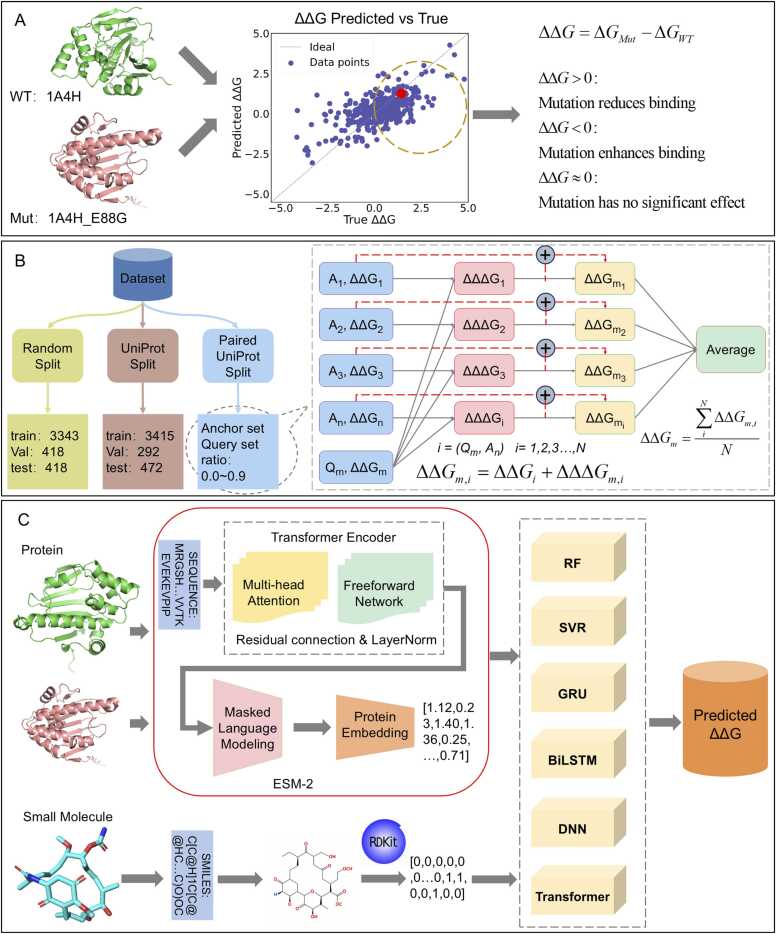
Workflow for relative binding free energy prediction using three dataset partitioning methods. (A) Method for calculating relative binding free energy changes ΔΔG induced by mutations. (B) Schematic diagram of data partitioning. (C) Workflow for feature construction and processing.

This study designed the following feature construction and processing process, as shown in Fig. 1.

(1) Protein sequence embedding extraction

We use the ESM-2 to learn the representation of the FASTA sequences of the wild-type protein and mutant protein in each data record and extract the protein sequence embedding from the last ESM-2 hidden layer before the output layer. The ESM-2 is based on the Transformer architecture and is pre-trained on a large-scale protein sequence database [30]. It can capture the contextual dependency and structural correlation of amino acid sequences. ESM-2 was the state-of-the-art protein language model, offering improved perplexity and downstream task performance over earlier models like ESM-1b. Its utility is evidenced by applications such as predicting mutation-induced protein stability [73].

(2) Protein embedding difference calculation

To effectively characterize the structural information changes before and after the mutation, we calculated the difference between the mutant and wild-type protein embedding vectors for each data record. The equation is as follows [74]:

(1) $$X_{protein}=R_{Mut}{-R}_{WT}$$

Where $R_{Mut}$ denotes the Embedding vector with dimension 1280 for the mutant protein, $R_{WT}$ denotes the Embedding vector with dimension 1280 for the wild-type protein, and $X_{protein}$ represents the feature characterizing the impact of the protein mutations. We also compare the conjoint fingerprints, where the embedding vectors are concatenated as follows.

(2) $$X_{protein}=[R_{Mut}{,R}_{WT}]$$

(3) Ligand feature encoding

We used the RDKit toolkit to convert the SMILES expression of the ligand into ECFP. This fingerprint is used to capture the local structure and functional group distribution information of the ligand as the input of molecular structure features. We employed ECFP4 with a radius of 2 and a fingerprint length of 1024 bits, which is a commonly adopted configuration balancing structural resolution and computational cost.

(4) Feature combination

We concatenate the protein difference embedding vector with the ligand ECFP to construct the final model input vector. The equation is as follows [75], [76]:

(3) $$X_{input}=[X_{protein}{,X}_{ligand}]$$

The dimensions of $X_{input}$ for subtracted and concatenated features are 2304 and 3584.

(5) Building and training the model

We selected six classic ML and DL models, including: Random Forest (RF), Support Vector Regression (SVR), Gated Recurrent Unit (GRU), Bidirectional Long Short-Term Memory (BiLSTM), Deep Feedforward Neural Network (DNN), and Transformer encoder (TF).

(6) Relative binding free energy prediction

We use the experimentally measured relative binding free energy (ΔΔG) data provided in the MdrDB dataset as the regression target for supervised learning of the model. The equation is as follows:

(4) $$\Delta \Delta G=\Delta G_{Mut}-\Delta G_{WT}$$

Where $\Delta G_{M\mathrm{ut}}$ and $\Delta G_{WT}$ are the binding free energies of mutant and wild-type proteins, respectively. The calculation method for ΔΔ*G* is shown in Fig. 1A.

To validate the mutation locations on the prediction performance, we classify the mutations into binding pocket, intermediate, and distal sites based on the distance between ligands and the mutated residues with distance bins (< 8 Å, 8 Å-15 Å, > 15 Å).

### 2.2 Data partitioning strategy

This study examined two typical data partitioning schemes to analyze the impact of the data set partitioning mode on the generalization performance of the protein-ligand binding free energy prediction model, as shown in Fig. 1B. These two partitioning strategies are random partitioning and UniProt-based partitioning.

(1) Random partitioning

We randomly divided all 4179 samples into training set, validation set and test set (train:3343, val:418, test:418) in a ratio of 8:1:1. This division method ensures that the distribution of proteins, ligands and mutants in each set is as close as possible to the original data set, which is used to evaluate the fitting ability and baseline performance of ML/DL methods under conventional settings.

(2) UniProt-based partitioning

We grouped the data according to the UniProt ID of the protein, giving priority to ensuring that samples related to the same protein do not appear in both the training set and the test set. We extracted UniProt IDs and randomly selected about 80 % of the UniProt IDs to construct the training set, and the remaining are then divided into 10 % for the validation set and 10 % for the test set. This strategy can effectively test the model's extrapolation ability in predicting new protein mutants, avoid information leakage, and reflect the real application scenario.

### 2.3 Anchor-query pairwise learning framework

To leverage the known states, we design a pairwise anchor-query partitioning. We include a sequential ratio (from 0.1 to 0.9) of known samples as the anchoring states to reveal the confidence intervals. By computing the relative-relative binding free energy (ΔΔΔ*G*) between anchor and query states, we can obtain a distribution of relative binding free energy of query states. The proposed anchor-query framework can be positioned as a domain-specific adaptation of pairwise learning, conceptually related to ΔΔΔ*G*-based computational mutagenesis methods. Previous studies emphasized the ranking of different ligands for one protein. Unlike generic pairwise or contrastive learning frameworks that compare two similar states, our strategy explicitly designates experimentally characterized states as anchors, thereby ensuring that predictions remain physically interpretable. It is specifically designed for mutation-dependent binding free energy prediction, using experimentally measured ΔΔ*G* values to calibrate predictions for uncharacterized queries. The anchor-query pairing method also has its limitations and may be constrained by the limited availability of reference data to serve as anchors.

In practical applications, we have collected mutation-induced binding free energy changes (ΔΔ*G*) for a limited set of reference samples. These curated reference data can be formally designated as anchor states. Given *N* anchor states, each query sample (representing an uncharacterized mutation) is systematically paired with all *N* anchors to generate pairwise comparisons. The free energy difference between anchor and query will be the new regression target as ΔΔΔ*G*. This approach yields *N* independent predictions for the query state, reflecting its relative binding affinity across multiple reference states. The predicted ΔΔ*G* can be recovered by adding ΔΔΔ*G*_*pred*_ with ΔΔ*G*_*anchor*_. The final predicted ΔΔ*G* for the query is derived as the ensemble average over the *N* anchor-specific predictions, enhancing robustness against single-reference biases.

(5) $$\Delta \Delta G_{query}=\sum \left(\Delta \Delta \Delta G_{pred,i}+\Delta \Delta G_{anchor,i}\right)/N$$

To validate this idea, we select the three most frequent UniProt IDs (Q13315, P00533, and P04637) as the initial test set (query set), and the rest as the training set. Then, we randomly extract parts of data from the test set at different ratios from 0.0 to 0.9 to form an anchor set, and merge it into the training set for model training. Finally, we evaluate its performance.

Collectively, these three strategies provide a comprehensive ground truth for analyzing the impact of data partitioning on prediction accuracy, model robustness, and the ability to improve performance using a limited set of reference samples. All partitioning is performed using randomly generated seeds 30 times, ensuring fair and reproducible comparisons of different model architectures. The additional introduction can be found in the SI for six classic ML/DL models: RF, SVR, GRU, BiLSTM, DNN, and the Transformer model.

The biological interpretation of our anchor-query framework is rooted in a comparative analysis of states. Given the known binding affinity of an anchor state, the objective is to predict the differential binding affinity to a query state. Determining the difference between two states is inherently more tractable than ab initio prediction of an absolute value. This is due to our proficiency in comparative learning, while generating a value without a reference presents a significantly greater challenge. Therefore, the model is trained to predict this affinity difference (ΔΔG) directly against the difference in the molecular representations (e.g., ESM2 embeddings) of the anchor-query pair. This establishes a direct mapping from representational divergence to a biophysical outcome.

### 2.4 Model evaluation metrics

We use four evaluation metrics to comprehensively measure the prediction ability of ML/DL models for protein-ligand binding relative free energy (ΔΔG), namely mean absolute error (MAE), root mean square error (RMSE), Pearson correlation coefficient, and Spearman correlation coefficient. These metrics can reflect the performance of the examined models in terms of error margin, outlier sensitivity, and correlation.

(1) MAE

The mean absolute error (MAE) measures the average level of absolute deviation between the model prediction value and the experimental observation value (ΔΔG) and is an important indicator reflecting the prediction accuracy. The calculation formula is as follows.

(6) $$MAE=1/n\sum_{i=1}^{n}\left|y_{i}-{\overset{\wedge }{y}}_{i}\right|$$

where $y_{i}$ denotes the experimentally measured relative binding free energy, ${\overset{\wedge }{y}}_{i}$ is the model prediction value, and n is the number of test samples.

(2) RMSE

The root mean square error (RMSE) is defined as the square root of the square differences between the predicted and the true value, placing greater emphasis on large prediction errors and making it more sensitive to outliers [77]. The calculation formula is as follows.

(7) $$RMSE=\sqrt{1/n\sum_{i=1}^{n}{\left(y_{i}-{\overset{\wedge }{y}}_{i}\right)}^{2}}$$

(3) Pearson correlation coefficient

The Pearson correlation coefficient measures the linear correlation between the model prediction value and the experimental observation value, with a range from −1 to + 1. Values closer to 1 indicate a stronger positive correlation. The calculation formula is as follows.

(8) $$r=\sum_{i=1}^{n}\left(y_{i}-\overset{¯}{y}\right)({\overset{\wedge }{y}}_{i}-\overset{\bar{\wedge }}{y})/\sqrt{\sum_{i=1}^{n}{\left(y_{i}-\overset{¯}{y}\right)}^{2}}\sqrt{\sum_{i=1}^{n}{\left(y_{i}-\overset{\bar{\wedge }}{y}\right)}^{2}}$$

where $\overset{¯}{y}$and $\overset{\bar{\wedge }}{y}$ denote the mean of the experimental value and the mean of predicted values, respectively.

(4) Spearman correlation coefficient

The Spearman correlation coefficient is used to measure the monotonic relationship between the ranks of predicted and experimental values, capturing both nonlinear and ordinal associations [78]. The calculation formula is as follows.

(9) $$\rho =1-6\sum_{i=1}^{n}d_{i}^{2}/n(n^{2}-1)$$

where $d_{i}$ denotes the difference in ranks between the experimental and the predicted values for the i-th sample.

For each model, all experiments were repeated 30 times independently, with each experiment using different random seeds to comprehensively account for uncertainties in both data partitioning and model training. All evaluation metrics are reported as mean ± standard deviation (mean ± SD), reflecting not only the average predictive performance of the models but also their stability and reproducibility.

The computing environment requires the ESM-2 model, the deep learning library torch 1.12.0, and the data analysis package Scikit-learn 1.0.2 and RDKit 2023.3.2. Python 3.7.16 is used for data analysis.

## 3 Results and discussion

### 3.1 Hyperparameter optimization

**Fig. 2:**
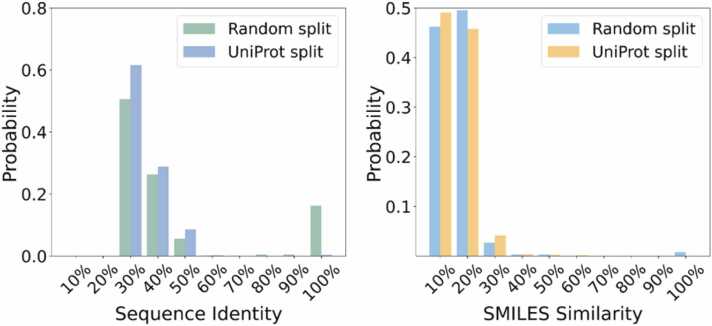
Sequence and SMILES similarity between training and test sets using random and UniProt-based splitting. All similarity values are represented as percentages.

In this study, the relevant results under the two strategies of random partitioning and UniProt-based partitioning are shown in Figure S1 and Figure S2. To further evaluate the stability and reliability of the results, we repeated the model 30 times and calculated the Pearson correlation coefficient of the model to identify the optimal model hyperparameters as shown in Figure S3 and Figure S4. ESM-2 achieves comparable performance in our task in comparison with ESMC and ProtT5 by using the same RF method (Figure S2). The method used to split data into training and test sets critically impacts the realistic assessment of a model's performance. As shown in Fig. 2, when proteins are split randomly, there is a high probability that proteins from the same family (e.g., homologous proteins with high sequence identity) will be distributed across both the training and test sets. Therefore, random splitting will likely produce overly optimistic results. UniProt-based splitting can separate proteins in the test set with low sequence identity. By enforcing a strict separation at the sequence level, this approach will lead to less data leakage. The model is forced to make predictions for truly novel entities, providing a much more rigorous and realistic test of its generalization capability.

First, under the random partitioning strategy, each model generally has a higher Pearson correlation coefficient. In detail, the performance of the RF is significantly improved in the early stage of the increase in the number of decision trees, but the gain tends to be flat after reaching a certain threshold, and the maximum tree depth parameter has only a slight positive effect. The SVR achieves the best performance when the regularization parameter C is moderate and the epsilon value is small, but its overall performance is still worse than that of the RF. Compared with deep learning models, moderately increasing the hidden layer dimension can generally improve model performance. However, setting too large a dimension may lead to overfitting or diminishing returns. Controlling the Dropout rate around 0.3 can effectively alleviate the overfitting problem. For BiLSTM, DNN, and Transformer architectures, simply increasing the number of network layers does not necessarily lead to sustained performance improvements, which suggests that the setting of network complexity needs to fully consider factors such as the scale of training data.

The difference is that under the UniProt partitioning condition, the model performance is much more sensitive to hyperparameters. Thus the Pearson correlation coefficient decreases overall and the variance increases. The increase in RF and SVR complexity does not bring stable benefits. In the cross-validation scenario, the optimal hyperparameters obtained under random partitioning are difficult to migrate directly. For neural network models, smaller hidden layer dimensions and low Dropout rates often lead to poor generalization capabilities. The results show that the optimal hyperparameters are highly dependent on the data distribution and partitioning strategy. Therefore, it is recommended to optimize hyperparameters for each evaluation scenario separately, and pay more attention to the generalization ability of the model, rather than just pursuing the maximization of performance on the validation set.

### 3.2 Comparison of feature combination strategies: concatenation and subtraction

**Fig. 3:**
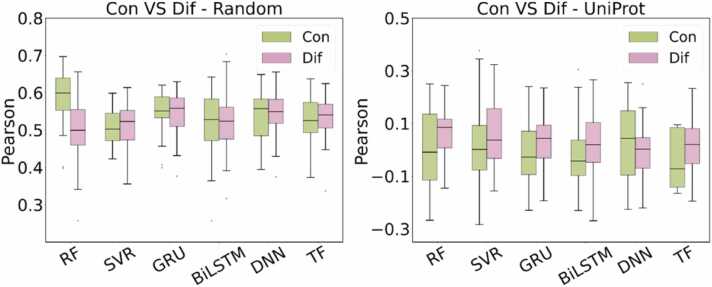
Comparison of Pearson correlation coefficients between concatenation and feature difference under random and UniProt-based partitioning.

In order to study the impact of different feature combinations on the model prediction performance, we compared the concatenation and difference feature methods under the two data partitioning conditions. The experimental results are shown in Fig. 3. The use of combining features difference can improve the Pearson correlation coefficient and has better prediction performance than the concatenation method. Under the random partitioning condition, three out of six models achieve higher Pearson coefficients using the feature difference than that of the concatenation method. Under the UniProt-based partitioning condition, when the test protein does not appear in the training set at all, the advantage of the feature difference is particularly prominent. The Pearson coefficients of the five out of six models under the feature difference are all higher than the concatenation, which fully proves that the difference combining can better capture the subtle effects of mutations and enhance the model's generalization ability. The differential feature representation outperforms the concatenated representation by capturing the differences in embeddings between wild-type and mutation-type peptides, enabling models to effectively leverage subtle distinctions. The results indicate that encoding molecular changes in the form of feature difference can achieve more stable protein-ligand relative free energy (ΔΔ*G*) predictions compared to concatenation. Therefore, the following evaluation adopts feature difference as the combining method.

### 3.3 Performance Analysis of random and UniProt partitioning

**Fig. 4:**
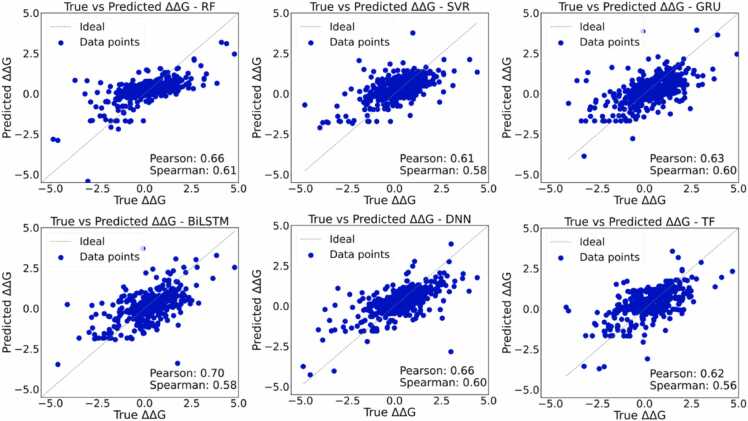
Correlation between the predicted and experimental ΔΔG values for random partitioning on the dataset. The unit of ΔΔG is kcal/mol.

To systematically evaluate the predictive capabilities of different modeling methods, we comprehensively compared the regression performance of two ML models (RF, SVR) and four DL models (GRU, BiLSTM, DNN, Transformer encoder) under two strategies: random partitioning and UniProt-based partitioning. The corresponding relationship between the predicted value and the experimental value of ΔΔ*G* of each model on the test set is shown in Fig. 4 and Figure S5.

Under the random partitioning condition, the prediction results of all models showed a moderate to high linear correlation with the experimental values. The DL models (especially BiLSTM and DNN) performed best overall, with the Pearson correlation coefficient reaching up to 0.70. The ML models, RF and SVR, also showed good predictive ability, with correlation coefficients ranging from 0.61 to 0.66. Although random partitioning yields apparently strong generalization across all model architectures in learning protein-ligand free energy relationships, these results likely represent optimistic estimates of true predictive capability due to inherent data leakage between training and testing partitions.

However, under the UniProt partitioning scenario, the predictive power of all models decreased significantly. As can be seen from the scatter plot, the alignment trend between the predicted and experimental values is significantly weakened, and the data points are dispersed. The Pearson and Spearman correlation coefficients of each model dropped below 0.32, and most were even close to zero. This result fully demonstrates the limitations of current methods in cross-protein generalization scenarios, that is, the transferability of models is limited when faced with unseen proteins or mutants. Further comparisons also revealed subtle differences among different model types. For example, BiLSTM and DNN show certain robustness advantages under both partitions, but under the UniProt partition, this advantage is significantly reduced, suggesting that even for complicated models, cross-protein sequence-function generalization is still challenging.

The performances of models using random partitioning and UniProt-based partitioning were compared by calculating the MAE, RMSE, Pearson correlation coefficient, and Spearman correlation coefficient of the test set. As shown in Table 1, we have summarized the prediction performance of six models under the random partitioning strategy. When the dataset is randomly partitioned, all models show low prediction errors and moderate correlation coefficients. Specifically, the DNN and GRU models have the lowest MAE, with corresponding RMSEs of 1.13 ± 0.12 and 1.16 ± 0.14, respectively as summarized in Table S1. The Pearson correlation coefficient and Spearman correlation coefficient of the two are both around 0.54, indicating that there is a moderate degree of consistency between the predicted values and the experimental values. The other four models (RF, SVR, BiLSTM, and Transformer encoder) also achieved similar results, showing relatively stable performance when the distribution of the training set and the test set is similar. The above results show that, under the independent and identically distributed scenario, all the models tested can effectively capture the relationship between protein-ligand characteristics and changes in binding free energy. Random partitioning may overestimate predictive performance.

**Table 1:** Performance evaluation of each model on the randomly partitioned and UniProt-based partitioned datasets.

	**Random**		**UniProt**	
	**Pearson**	**Spearman**	**Pearson**	**Spearman**
**RF**	0.49 ± 0.09	0.53 ± 0.05	**0.06 ± 0.10**	0.07 ± 0.11
**SVR**	0.51 ± 0.06	0.52 ± 0.04	0.05 ± 0.14	**0.08 ± 0.14**
**GRU**	**0.54 ± 0.06**	**0.53 ± 0.04**	0.03 ± 0.09	0.02 ± 0.12
**BiLSTM**	0.52 ± 0.09	0.51 ± 0.03	0.02 ± 0.11	0.01 ± 0.13
**DNN**	0.54 ± 0.07	**0.53 ± 0.04**	−0.01 ± 0.10	−0.00 ± 0.13
**Transformer**	0.53 ± 0.06	0.52 ± 0.03	0.01 ± 0.11	−0.01 ± 0.13

In contrast, the results under the UniProt-based partitioning strategy, the model performance has dropped significantly. Given that our test set is composed of UniProt protein mutations that have never appeared in the training set, the prediction correlation is expected to be reduced. The MAE of all models has increased to the range of 0.99–1.31, and the RMSE has also generally increased. In particular, the Pearson and Spearman correlation coefficients of all models have dropped to near zero, and even negative values have appeared. This sharp decline in correlation indicates that when the model faces a completely new protein, its generalization ability is severely limited and its predictive ability is significantly reduced. The above results highlight the challenges of predicting changes in binding free energy of new protein targets, and also illustrate the need to address the problem of distribution shifts between training and test sets by improving feature representation and model structure.

### 3.4 Evaluation of performance with gradual introduction of reference data

**Fig. 5:**
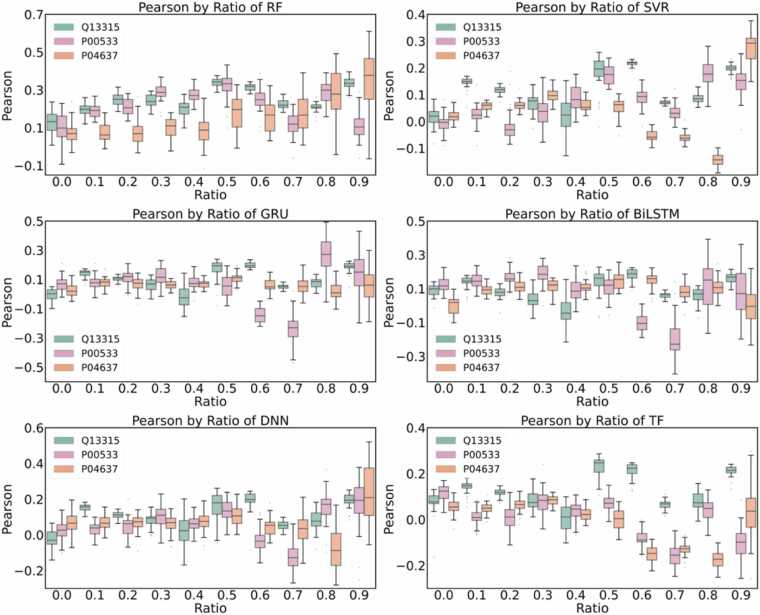
Pearson coefficient changes with simple gradual introduction of reference data with ratio from 0 to 0.9 for three systems of Q13315, P00533 and P04637. The reference data is directly included in the training dataset without constructing anchor-query pairs.

To explore the effect of changing the amount of reference data on the accuracy of protein-ligand relative free energy prediction, we also set up a third division method and conducted systematic experiments on the three UniProt proteins with the highest frequency in UniProt, namely Q13315, P00533, and P04637. The selected proteins represent three functional classes. Q13315 corresponds to the DNA-damage checkpoint kinase ATM (a PIKK-family nuclear serine/threonine kinase) with 304 proteins, P00533 corresponds to the receptor tyrosine kinase EGFR with 170 proteins, and P04637 corresponds to the tumor-suppressor TP53 (a transcription factor) with 162 proteins, thereby spanning kinase, membrane receptor, and transcription-factor categories. The relative binding free energy ranges for Q13315, P00533 and P04637 are as follows: −1.561 to 3.078 kcal/mol, −4.042 to 2.31 kcal/mol, −2.605 to 1.545 kcal/mol, respectively. We used Q13315, P00533, and P04637 as test data, and the rest as train data. Then, we randomly extracted the data from the test in the proportions of 0.0, 0.1, 0.2, 0.3, 0.4, 0.5, 0.6, 0.7, 0.8, and 0.9, and introduced the selected samples into the training data. Using the same hyperparameters, we predict the relative binding free energy (ΔΔG) of protein-ligand under the condition that parts of the samples are known. As shown in Fig. 5, when the anchor ratio is 0.0, the Pearson correlation coefficients of all models approach zero, which means that the model is difficult to achieve effective generalization for new proteins. The Pearson correlation coefficients of each model increase in a step-by-step manner, as the anchor data in the training set gradually increases. Even with the introduction of a very small amount of anchor data (10 %), the prediction performance of all models has improved as shown in Fig. 5. With the increase of the anchor data ratio, the accuracy of the model continues to improve. When the anchor ratio is higher than 0.3, the correlation coefficients of models such as RF, BiLSTM, and DNN reach to their full capacity with coefficients around 0.3.

This result fully proves that in the absence of reference data, the prediction task of binding affinity prediction of new proteins still faces great technical challenges. The results are consistent with the ligand binding results of CASP16, where the best correlation coefficient is 0.42 for the absolute binding affinity prediction [79]. When 10–20 % reference data was introduced, the model prediction performance was improved, among which the BiLSTM and DNN models performed more prominently, and the correlation coefficients are increased with only a medium anchor ratio. When the anchor ratio exceeded 0.7, all models, including RF, SVR, and TF, tended to be stable, and their correlation levels were similar to the results of random partitioning experiments. The results based on the hyperparameters of UniProt partition optimization show similar prediction accuracy (Figure S6). In our study, RF provides the best results than other deep learning models. RF is an ensemble learning method and provides effective learning when the data is not large, while deep learning is a data-greedy method and cannot fully exploit its capacity by using the current dataset and input molecular representations. But the performance is still far from satisfactory. A significant limitation for practical application is the method's reliance on reference data, which is often unavailable in the early stages of drug discovery projects. Our anchor-query approach can only be deployed after accumulating several critical data.

### 3.5 Evaluation of performance of the model with query-anchor partitioning

Though simply introducing reference data into the training dataset can improve the prediction performance, the performance still cannot satisfy the application. Therefore, we propose a new way to include the reference data in the training process. We build each unknown sample (query) with the reference samples. The reference samples will act as the anchor to make the prediction around their neighbors. To validate the query-anchor partitioning strategy, we follow the same UniProt-based partitioning approach, but the randomly extracted data were named the anchor set, and the remaining test was named the query set.

**Fig. 6:**
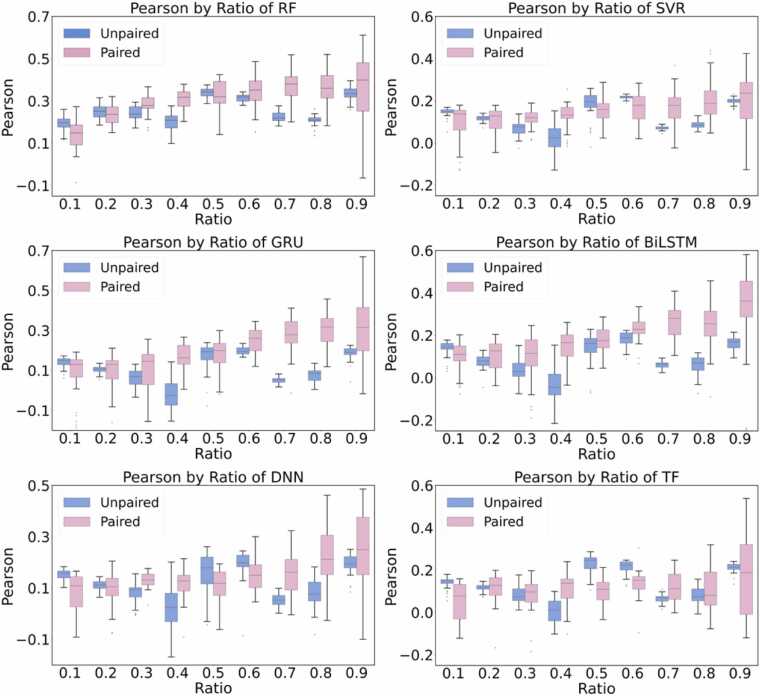
Pearson coefficients comparison between the simple gradual introduction of reference data into the training set and the construction of anchor-query pairs. A simple gradual introduction of reference data is labeled as Unpaired. The construction of the anchor-query pair is labeled as Paired.

The anchor-query paired strategy consistently outperforms the unpaired approach across all models, particularly at higher ratios (e.g., 0.7–0.9). Specifically, for RF and SVR models, paired data consistently demonstrate superior Pearson correlations, reflecting enhanced predictive reliability compared to the unpaired approach. Notably, RF exhibits significant improvements at higher ratios, underscoring its ability to leverage structured relationships within paired data effectively. For GRU and BiLSTM, paired data yields a steeper improvement in correlation compared to unpaired data, indicating that these models benefit from relational constraints. For DNN and TF, the paired strategy performs worst at intermediate ratios (0.5 and 0.6). This result implies that well-designed pairing provides more meaningful learning than the simple incremental addition of data in the training set. Nevertheless, the overall performance is still far from satisfactory, underscoring that mutation-dependent binding free energy prediction has not yet surpassed the fundamental limitations observed in CASP16.

From the above analysis, we can get the following conclusions. First, in the absence of anchor data, the generalization ability of various algorithm models on all tested proteins is suboptimal, and this phenomenon is unrelated to the algorithm. Second, the results demonstrate that even a modest incorporation of reference data (as low as 20 %) significantly enhances the predictive accuracy of all models. This underscores the critical role of reference data in improving model robustness. Third, the prediction of relative binding free energies remains a further refinement requirement despite improvements. Current methodologies exhibit limitations, particularly in scenarios with sparse training data. Fourth, models trained using the anchor-query partitioning strategy exhibit superior performance compared to alternative approaches. However, the performance ceiling of this strategy, as observed at a high reference ratio (0.9), only matches that of random partitioning. This finding highlights the importance of evaluation frameworks grounded in real-world application scenarios to enhance model generalizability. Random data partitioning yields performance metrics that reflect the maximum achievable prediction accuracy for a given model. Under conditions of sparse training data, predictive performance is fundamentally constrained by this upper bound. Rational data utilization, such as constructing anchor-query pairs, can provide modest improvements in prediction accuracy. The maximum correlation achieved under this framework (Pearson ∼ 0.3 with 20–30 % anchors) is still modest, underscoring the limited cross-protein transferability of current sequence-based approaches. This limitation highlights the need for future work combining anchor-query pairing with structure-aware features or physics-based methods to further improve predictive accuracy.

### 3.6 Comparison with physics-based method

**Fig. 7:**
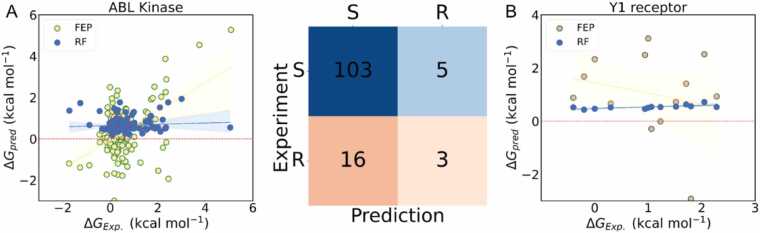
Prediction comparison of RF and FEP on ABL kinase and neuropeptide Y Y1 receptor. (A) Left: scatter plot of predicted versus experimental ΔΔ*G* values for ABL kinase. Middle: confusion matrix of resistant (R) versus susceptible (S) classification using a 1.36 kcal/mol threshold. (B) validation on neuropeptide Y Y1 receptor (UniProt ID: P25929, PDB ID: 5ZBQ), showing consistency of RF and FEP predictions across structural contexts.

Accurate prediction of relative binding free energy is still a challenge for both deep learning and physics-based methods. We compared our results with those of the well-established physics-based methods, including Free Energy Perturbation (FEP) [80] and thermodynamic integration (TI) [81]. The predictions were carried out on the same protein family of ABL kinase. Our results achieved comparable accuracy to those of FEP as shown in Fig. 7. The Root Mean Square Error (RMSE) of the binding free energy was 0.87 kcal/mol, which was lower than the 1.11 kcal/mol obtained by FEP [80]. Additionally, we compared the classification performance using the same criterion of 1.36 kcal/mol, which corresponds to a 10-fold change in IC50 affinity, to distinguish between susceptible and resistant mutation types. Our method achieved an accuracy of 0.83, which was lower than the 0.89 of FEP. The Area Under the Curve (AUC) was 0.62, which was higher than the 0.56 of TI [81]. Our proposed method strives to reach the chemical accuracy of physics-based methods. To further substantiate the evaluation, we extended our analysis to the human neuropeptide Y Y1 receptor (UniProt ID: P25929, PDB ID: 5ZBQ) [82]. As shown in the Fig. 7B, FEP and RF predictions for this system yield the Spearman coefficients as −0.137 and 0.791. The RMSE and classification accuracy of RF are 0.937 and 0.615. This consistency across two structural contexts (ABL kinase and the Y1 receptor) reinforces the conclusion. The relative trends between FEP and RF remain aligned, underscoring the necessity of integrating reference anchors or structural features for meaningful predictive generalization.

Our study shows that the data partitioning strategy directly determines the potential accuracy that can be achieved in the prediction of the relative free energy (ΔΔ*G*) of protein-ligand binding. Under random partitioning, the model performs well, which shows Pearson coefficients above 0.5. However, this high accuracy overestimates the generalization ability of the model in practical applications. The higher coefficients are due to the overlapping samples between the training set and the test set. When a partition based on UniProt or anchor-query is used, which is closer to the real scene, the prediction ability of all models is significantly reduced, exposing the problem of the limited generalization ability of existing methods. In the absence of reference data, all models fail to obtain meaningful prediction results. When parts of known samples are included in the training set, the performance will be improved. Based on this finding, we propose an anchor-query strategy. The experiments further show that the prediction accuracy can be improved even if only 20 % of the reference data is introduced. Therefore, the accuracy of protein-ligand binding free energy prediction is greatly limited by the data partitioning method. If some of the reference data is available, we can construct the anchor-query pairs to further improve prediction performance. The prediction of relative binding free energy remains in its nascent stages. Caution is warranted in interpreting these results, as the higher Pearson coefficients reported in prior studies were derived using random partitioning, which may not fully reflect real-world applicability.

### 3.7 Structural analysis on mutation locations

**Fig. 8:**
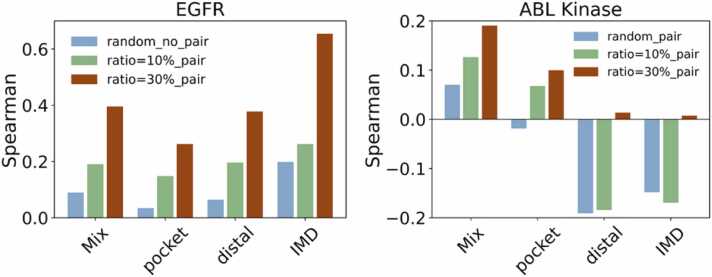
Spearman coefficients for the different mutation locations in two protein families. The mutation location is classified as binding pocket, intermediate (abbreviated as IMD), and distal site (abbreviated as distal) based on the distance as < 8 Å, 8 Å-15 Å, and > 15 Å.

We evaluated the impact of mutation location on the prediction performance by using two protein families, EGFR and ABL kinase. The results obtained under UniProt-based partitioning already reflect family-dependent effects: larger protein families such as Epidermal growth factor receptor (EGFR with UniProt ID: P00533) with 170 proteins are more frequently represented in the training set, which likely contributes to improved generalization within those families compared to small or sparsely sampled families with 127 ABL kinase proteins (UniProt ID: P00520 and P00519). Prediction performance also varies based on the mutation locations. Mutations within the binding pocket result in worse prediction accuracy than those elsewhere (Fig. 8), suggesting that these residues play a more critical role in determining binding affinity.

As shown in Fig. 8, the results differ between protein systems. For P00533 (EGFR), binding pocket mutations show the lowest correlations, while distal and especially intermediate (IMD) mutations achieve higher Spearman values, particularly when anchor–query pairing is applied (ratio = 30 % anchors, IMD > 0.6). For the mutation G719S+T790M on the binding with Gefitinib in EGFR, the best predicted binding energy is 0.435 while the experimental value is 0.48. But the predicted and experimental binding energies are −0.07 and 0.313 for the mutation of T790M+L858R. It highlights a potential boundary of our approach.

For the paired system P00520/P00519, the introduction of anchors provides only marginal improvements. Pocket mutations are generally more challenging to predict due to highly localized structural perturbations that sequence embeddings fail to capture. Prediction performance is protein-family dependent, with some systems (e.g., P00520/P00519) showing limited recoverability even with anchor support. The predicted binding energies for the important mutations T315I and E255K in ABL bound to Gleevec are 1.782 and 0.838, while the experimental value is 2.306 and 1.491. The ability to accurately rank the destabilizing effects of different mutations (e.g., predicting that T315I is more detrimental than E255K in ABL) is a powerful application. However, it is important to note that the accuracy of these predicted values is not sufficiently high, indicating that further improvements to the method are necessary.

Our anchor-query framework is indeed conceptually aligned with pairwise learning paradigms, which are central to many relative binding energy ΔΔ*G* prediction methods. In our approach, the model learns to predict the difference in relative binding affinity (ΔΔΔ*G*) between a 'query' and an 'anchor' complex. This is analogous to how pairwise learning is used to rank ligands relative to one another. The key extension in our work is the application of this pairwise concept not only within a single protein system (UniProt-based splitting) but also across different proteins (random splitting).

### 3.8 Limitations of our study

The findings reported here should therefore be interpreted in the context of these uncertainties of experimental measurements from the dataset and the unbalanced data.

First, a high-quality dataset is the critical factor for the model training. The adopted dataset, MdrDB, integrates mutation-dependent binding affinity data from multiple public sources. The data errors exist due to the differences in assay types, experimental protocols, and reporting standards. Although this provides broad coverage of protein–ligand systems, it also introduces several potential sources of bias. Binding and inhibition measurements differ across assay formats, laboratories, and conditions. Since detailed metadata (e.g., replicates, error margins) are often unavailable, the true level of experimental noise cannot be quantified, which may obscure the upper limit of model accuracy. We acknowledge that the absence of raw experimental error bars remains a limitation of the present work. We view the incorporation of higher-quality benchmark datasets, ideally with curated experimental data and explicit uncertainty estimates, as an essential direction for future work. Such datasets would allow us to better disentangle intrinsic experimental variability from true model performance.

Second, sample imbalance is another issue for performance. Large protein families (e.g., kinases) are better represented, whereas smaller or rarer families have only a few mutations. This imbalance can inadvertently favor models that generalize within well-sampled families, while underestimating the difficulty of extrapolation to underrepresented proteins. The dataset only covers a limited number of proteins and ligands, which introduces variability that may not be captured by sufficient samples.

Third, our method employed ESM-2 embedding as the molecular representation for protein sequences. However, the absence of structural information might restrict the performance. Thus, we verified the performance by constructing molecular graphs between protein pockets and ligands. The results indicated that the graph convolutional network (GCN) using the molecular graph achieved a Pearson correlation of 0.64 (Figure S7), which was higher than the 0.54 obtained for our ESM-2 embeddings. Our results indicate that the ESM-2 embeddings may not fully substitute for explicit structural descriptors when modeling mutation effects on binding free energy. A hybrid strategy could combine the strengths of both paradigms, for example, by using physics-based ΔΔ*G* calculations to generate anchor references or to regularize ML models, thereby guiding them toward physically plausible predictions. Such an approach may mitigate overfitting to dataset-specific biases and enhance transferability across diverse protein families. While this integration is beyond the scope of the current work, we consider it a promising avenue for future research.

## 4 Conclusion

This study comprehensively evaluated the impact of data partitioning strategies on the prediction accuracy of protein-ligand binding relative free energy (ΔΔ*G*) and revealed the upper limit of the performance that the models can achieve under different partitioning scenarios. The results show that although traditional random partitioning can obtain higher prediction correlation, given the highly similar distribution of the training set and the test set, this strategy may overestimate the generalization ability of the model in real systems. Based on UniProt partitioning, the correlation coefficient and accuracy are reduced, and the model's generalization ability for unknown proteins and mutants is still limited. In the absence of prior information, existing models generally have difficulty in achieving rational generalization. The analysis shows that a small amount of reference data (anchor set) can greatly improve the prediction accuracy when applied to new protein systems. However, only by using the known data in a reasonable and effective manner can we further enhance the prediction performance of the models. As evaluated, simply incorporating the known data into the training set can bring about some improvement, yet the improvement is limited. Pairing the known data with the unknown data will further boost the prediction performance. Our proposed anchor-query partitioning plays a key role in improving model robustness and extrapolation performance. The experimental results highlight the significance of reasonable dataset partitioning for objectively evaluating model performance and promoting the practical application of algorithms.

## Code and data availability

The example scripts presented in this study are made available at:

https://github.com/AIMedDrug/Data-Partitioning-Strategies.

The original dataset is obtained from https://quantum.tencent.com/mdrdb/

## Supplementary data

Supplementary data to this article can be found online.

## Funding

This work was supported by the 10.13039/501100001809National Natural Science Foundation of China (62373172, 22003020, and 12074151), the National Guidance Fund on Developing Local Science and Technology for Sichuan Province (No. 2023ZYD0167), the 10.13039/501100004608Natural Science Foundation of Jiangsu Province (BK20191032), and Changzhou Sci. & Tech. Program (CJ20200045).

## CRediT authorship contribution statement

**Dawei Zhang:** Validation, Investigation. **Lei Xu:** Visualization. **Xiaojun Xu:** Software, Formal analysis. **Shan Chang:** Supervision, Resources. **Liangxu Xie:** Writing – review & editing, Resources, Project administration, Conceptualization. **Guoming Bao:** Writing – original draft, Investigation, Data curation.

## Declaration of Competing Interest

The authors declare no competing interests.
