# Supplementary material for: Evaluating data partitioning strategies for accurate prediction of protein-ligand binding free energy changes in mutated proteins
Source: Comput Struct Biotechnol J. 2025 Oct 14;27:4418–30. doi: 10.1016/j.csbj.2025.10.020 (PMC12569818; doi:10.1016/j.csbj.2025.10.020)
Supplement: Supplementary file 1 — Supplementary material [file mmc1.docx]

# Evaluating Data Partitioning Strategies for Accurate Prediction of Protein-Ligand Binding Free Energy Changes in Mutated Proteins

Liangxu Xie, Guoming Bao, Dawei Zhang, Lei Xu, Xiaojun Xu, Shan Chang

Institute of Bioinformatics and Medical Engineering, Jiangsu University of Technology, Changzhou 213001, China

## Computational methods

Random Forest (RF) is a machine learning algorithm based on ensemble learning. It improves the accuracy and robustness of the model by constructing multiple decision trees and combining their prediction results. This study uses the random forest regression algorithm, combined with ESM-2 feature difference and ECFP fingerprint features, to predict the relative free energy (ΔΔG) of protein-ligand binding. Its prediction is achieved by averaging the output results of multiple trees:

where denotes the prediction result of the *i*-th tree, $N$is the total number of decision trees, and $\mathop{y}\limits^{\wedge}$ is the predicted relative free energy (ΔΔG) value.

Support Vector Regression (SVR) is a regression method based on the support vector machine (SVM) theory. By constructing an optimal regression hyperplane , SVR can effectively capture the complex relationship between free energy and molecular characteristics in the protein-ligand system. Most of the sample points will fall within the width interval band centered on the hyperplane (part in the figure$wx+b=\pm\varepsilon$). The optimization goal is to minimize the norm of the weight vector to the greatest extent ($\left\|w\right\|^{2}$), thereby avoiding overfitting of the model, and adopting an insensitive loss function to impose penalties only on sample points whose predicted values significantly deviate from the interval band. This ensures the sparsity of the model and improves its robustness to abnormal samples.

Gated Recurrent Unit (GRU) is an improved recurrent neural network structure. As a simplified variant of LSTM, GRU effectively controls the flow of information through a gating mechanism, solving the gradient vanishing or exploding problem of traditional RNN when processing long sequences. Its basic calculation is as follows:

Update Gate:

Reset the gate:

Candidate hidden states:

Hidden state update:

where $x_{t}$ denotes the input features, $\sigma$ is the sigmoid function, represents the element-wise product, and $W,U,b$ denotes the learnable parameters.

Bidirectional Long Short-Term Memory (BiLSTM) consists of two LSTM networks, forward and reverse, which can better capture the bidirectional feature relationship of the sequence. The forward LSTM processes the input sequence in chronological order (fromto) and generates the corresponding hidden state (such asto); the reverse LSTM processes the sequence in reverse order (fromto) and generates another set of hidden states. The network combines the hidden states of the two directions by splicing or weighting to form the complete output of BiLSTM, and passes it to the subsequent Feedforward layer or Backpropagation layer for further processing to predict the relative free energy of protein-ligand (ΔΔG).

Deep Neural Network (DNN) is a multi-layer neural network structure that learns the nonlinear relationship between protein embedding features (X1-X2) and ECFP fingerprint features through multi-layer nonlinear transformations, and achieves regression prediction of binding free energy from high-dimensional data. The neural network architecture employed in this study can be described as follows:

where $h^{(l)}$denotes the activation output of the $l$-th layer, $\sigma^{(l)}$ is the nonlinear activation function of this layer, $W^{(l)}$ is the weight matrix, $b^{(l)}$ is the bias term, and $L$ is the total number of layers in the network.

The final output layer is linear:

$$\mathop{y}\limits^{\wedge} =W^{(L+1)} h^{(L)} +b^{(L+1)}$$

The Transformer encoder model is based on the self-attention mechanism and is capable of capturing the long-range dependencies within protein embedding features and ligand ECFP fingerprint features. In this study, the Transformer treats the spliced protein-ligand feature sequence as the input sequence and effectively predicts the relative free energy (ΔΔG) through the multi-head attention mechanism. Its mathematical formulation is given by:

where $Q$ denotes the query, $K$ denotes the key, $V$ denotes the value, and $d_{k}$ is the dimension of the query or key.

The Transformer encoder model employs multi-head attention to project the input into multiple subspaces, allowing the model to simultaneously focus on information at different positions and feature dimensions in the sequence. The position encoding then injects sequence order information into the model through a sinusoidal function, while the feedforward network is position-based with a ReLU activation function, which enables the model to fully understand the sequence structure information and significantly improves the accuracy of experimental predictions.

**Graph Construction for GCN-Based ΔΔG Prediction**

To further capture the spatial organization of protein-ligand complexes beyond sequence embeddings, a graph-based representation was constructed for both wild-type and mutant systems. Each graph encodes atomic-level interactions within and between protein and ligand components, allowing the Graph Convolutional Network (GCN) to capture structural and physicochemical dependencies relevant to mutation-induced binding free energy changes (ΔΔG).

**(1) Node definition and features.**

Each atom in the protein-ligand complex was treated as a graph node. Node features were derived from both atomic and contextual attributes. The atomic type was first inferred and categorized into nine elemental groups: {C, N, O, S, P, H, METAL, HALO, X}, where “METAL” represents metallic elements (e.g., Zn, Mg, Fe) and “HALO” represents halogens (Cl, Br, I, F). A one-hot encoding of this category was concatenated with three binary indicators: (i) whether the atom belongs to the protein, (ii) whether it belongs to the ligand, and (iii) whether it is part of the protein backbone (atoms N, CA, C, O). In addition, three graph-topological attributes—normalized total degree, edge-type-specific degree, and mean edge distance—were appended to form the final feature vector for each node. This representation integrates both chemical identity and spatial connectivity into a unified atom-level descriptor.

**(2) Edge construction.**

Edges were generated based on pairwise Euclidean distances between atomic coordinates. Three distinct edge types were defined according to molecular origin: prot_intra (protein-protein), sm_intra (ligand-ligand), and inter (protein-ligand). Empirically determined cutoffs were used for edge creation: 5.0 Å for protein-protein and protein-ligand pairs, and 4.0 Å for ligand-ligand pairs. For each valid atom pair within the cutoff, bidirectional edges were added with an edge weight defined as the inverse of the interatomic distance (1 / [d + 10⁻⁵]). This ensured that closer atomic pairs contributed more strongly to message passing during GCN propagation.

**(3) Graph normalization and features aggregation.**

The degree-based features were normalized using the $\log(1+x)$ transformation to mitigate scale imbalance across different node types. For nodes with multiple edges, the mean of the connecting edge distances was computed to represent the local density around that atom. The final node feature vector combined chemical identity (elemental one-hot encoding) and structural topology (degree and distance statistics), resulting in a 17-dimensional input feature vector.

**(4) Graph pair formation for ΔΔG learning.**

For each protein-ligand system, a wild-type (WT) and its corresponding mutant (MT) graph were constructed following the same procedure. These paired graphs were then input into a Siamese GCN model that independently encoded each structure and learned the difference between their embeddings. The concatenation of the wild-type and mutant embeddings, together with their difference vector, was passed to a regression head to predict the experimental ΔΔG.

This graph construction process allowed the GCN to learn fine-grained topological and spatial relationships while maintaining chemical interpretability. The design enables the model to infer mutation-induced perturbations directly from atom-level connectivity patterns, without requiring predefined structural descriptors or handcrafted features.

## Supporting Figures


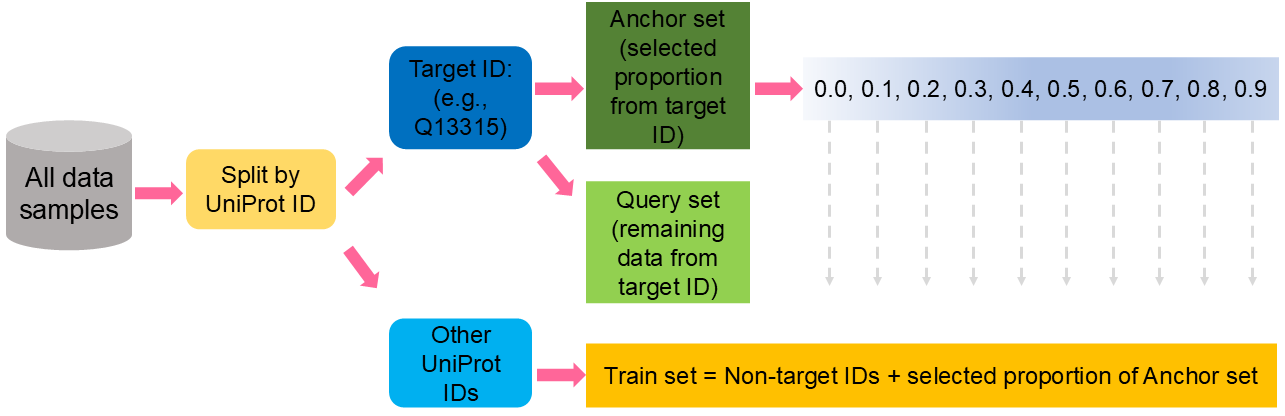


**Fig. S1** The proposed workflow of gradually introducing the reference data split from the targeted UniProt into the training set.


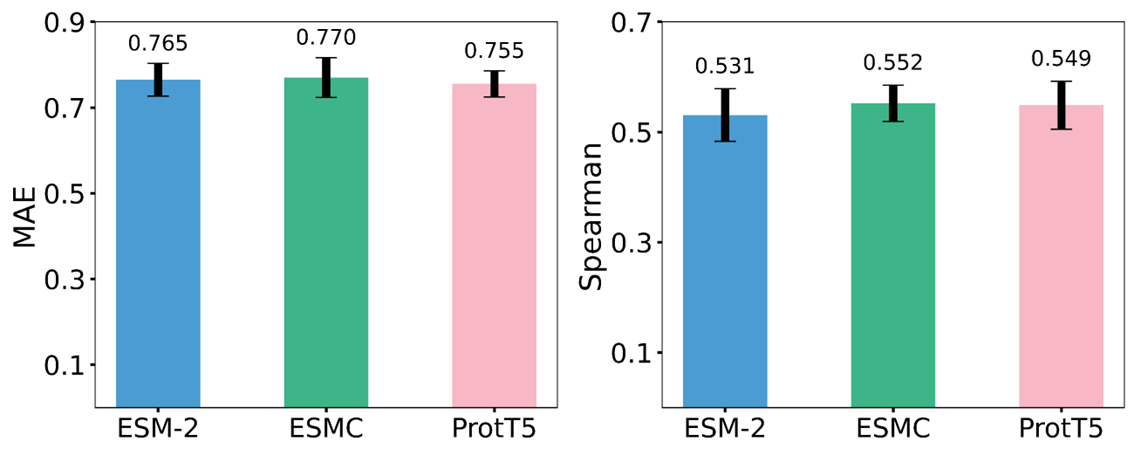


**Fig. S2** Comparison of protein language model embeddings using protein language model ESM-2, ESMC, and ProtT5 for RF-based ΔΔG prediction.


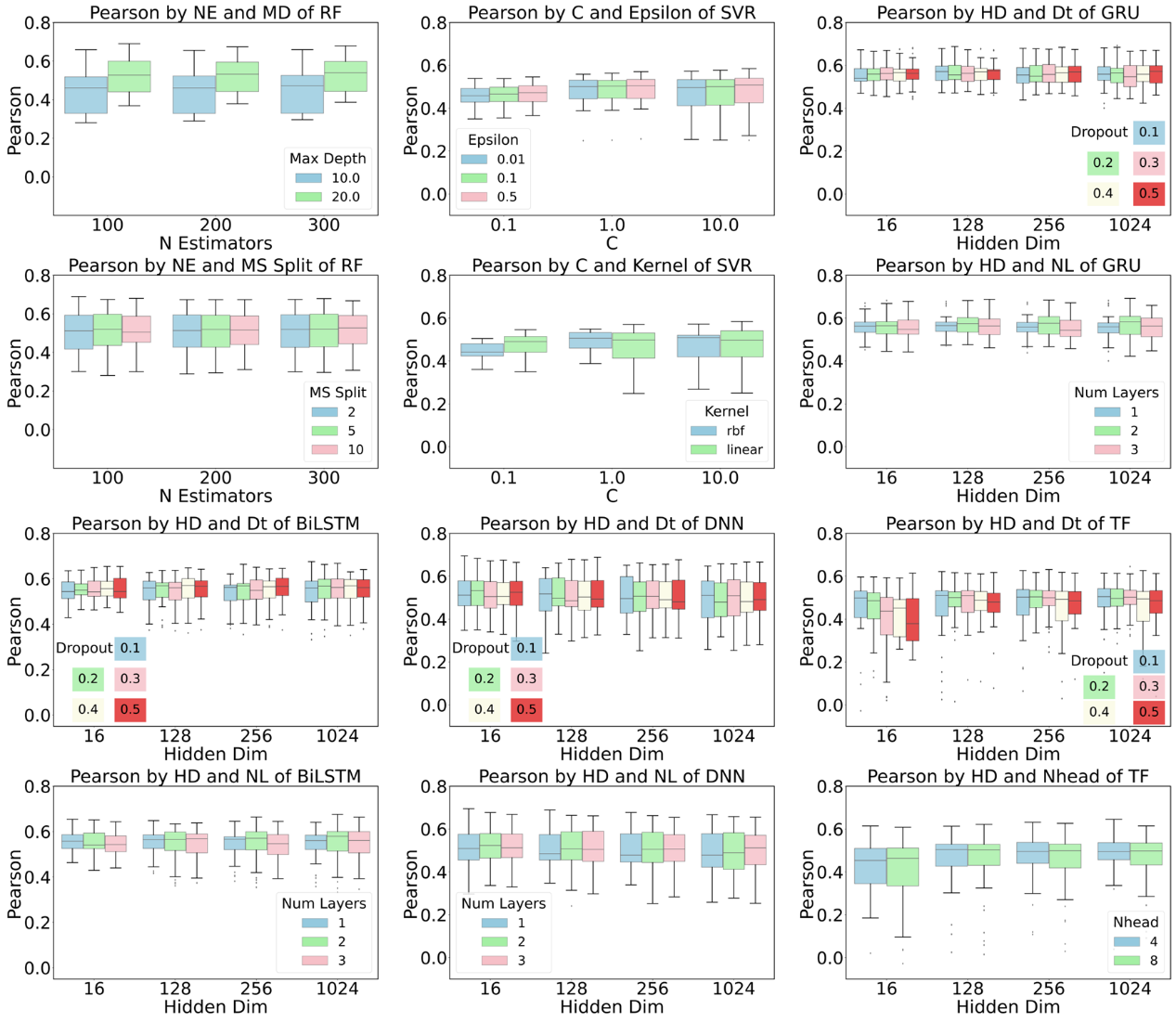


**Fig. S3** Distribution of Pearson correlation coefficients for each model under different hyperparameter settings on the randomly partitioned dataset. For clarity, Number of Estimators, Maximum Depth, Hidden Dimension, Dropout, Minimum Samples Split, Num Layers are abbreviated as NE, MD, HD, Dt, MS and NL.


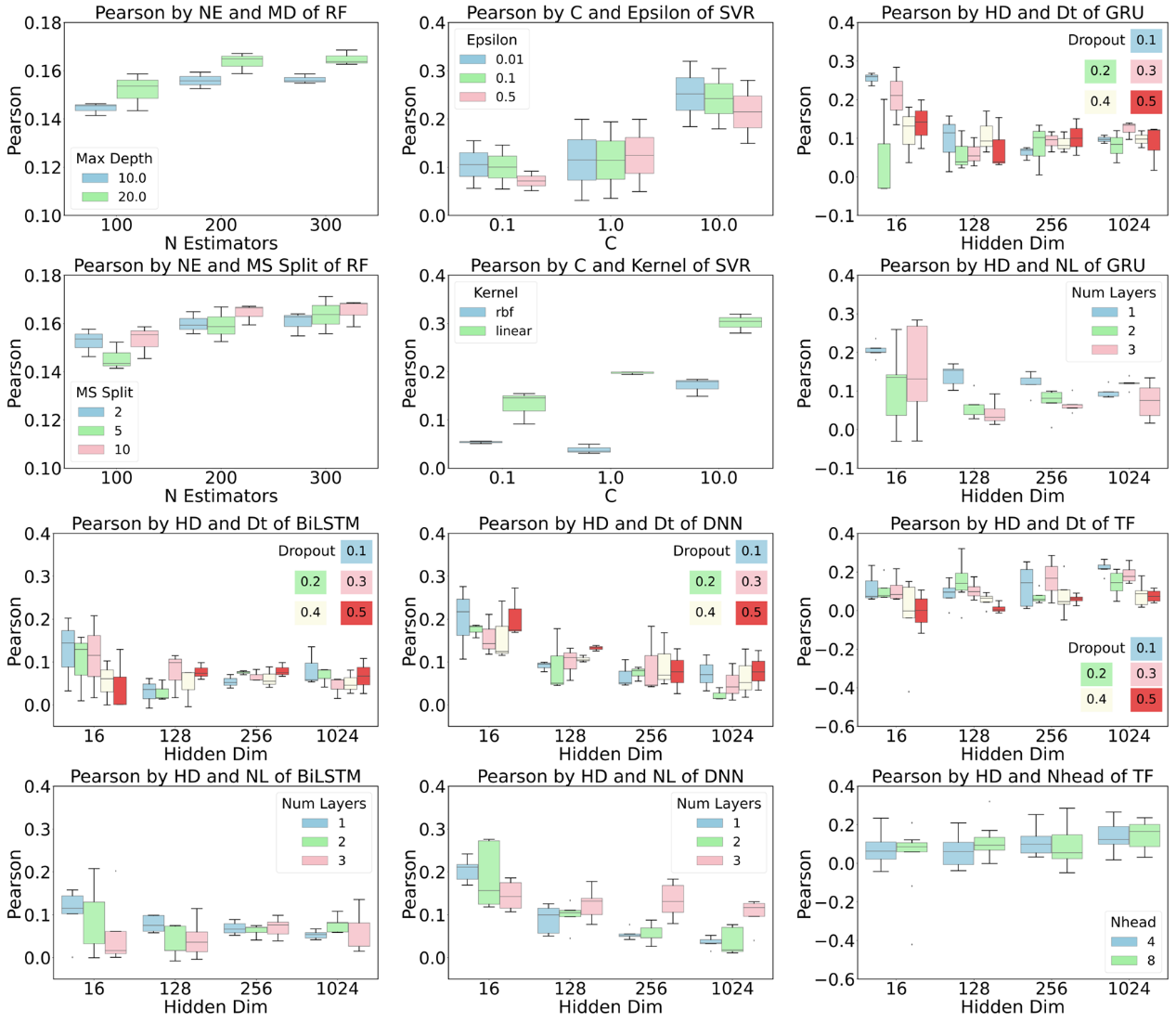


**Fig. S4** Distribution of Pearson correlation coefficients for each model under different hyperparameter settings on the UniProt-partitioned dataset.


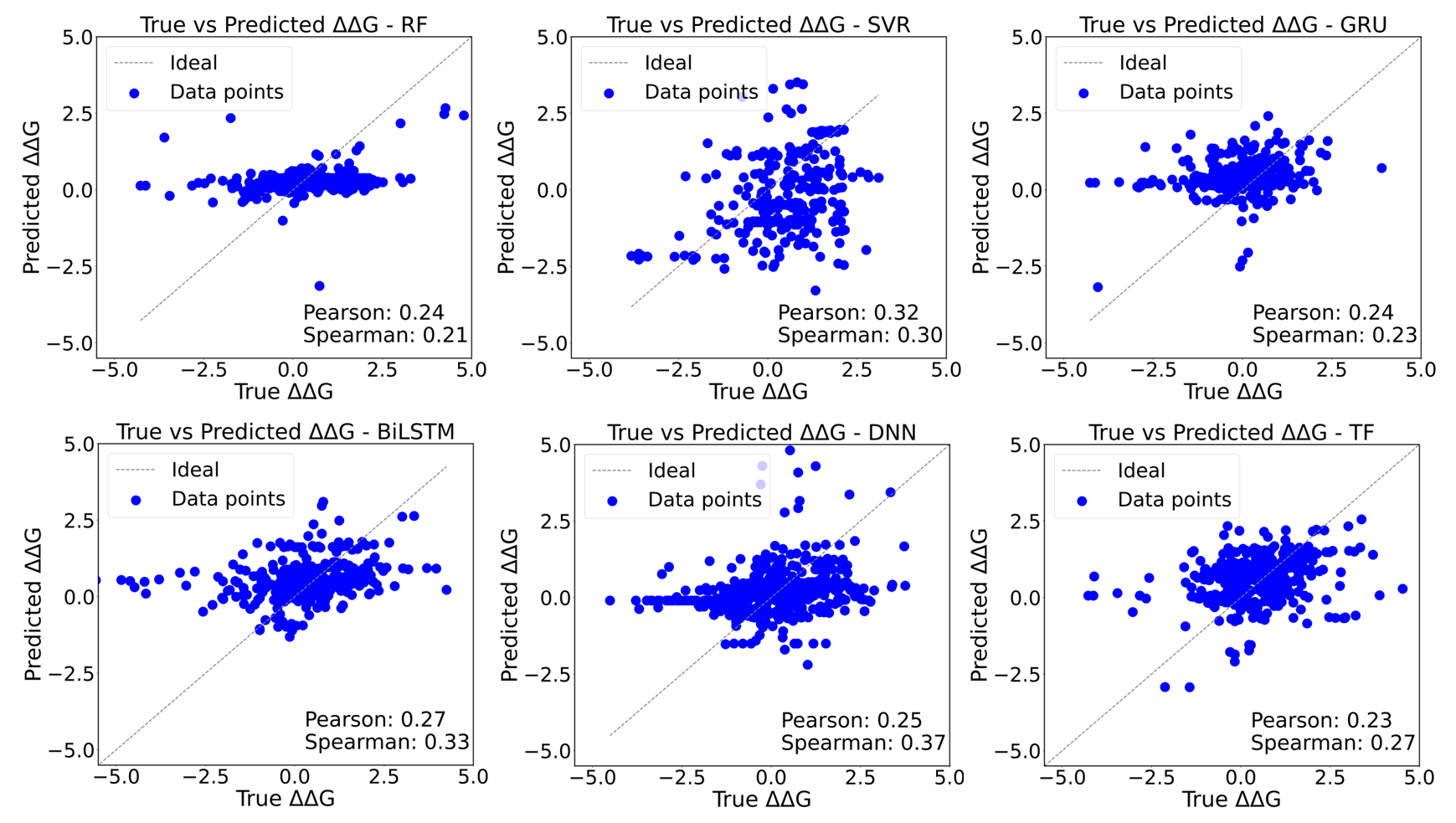


**Fig. S5** Correlation between predicted and experimental ΔΔG values on the UniProt-based partitioning dataset.


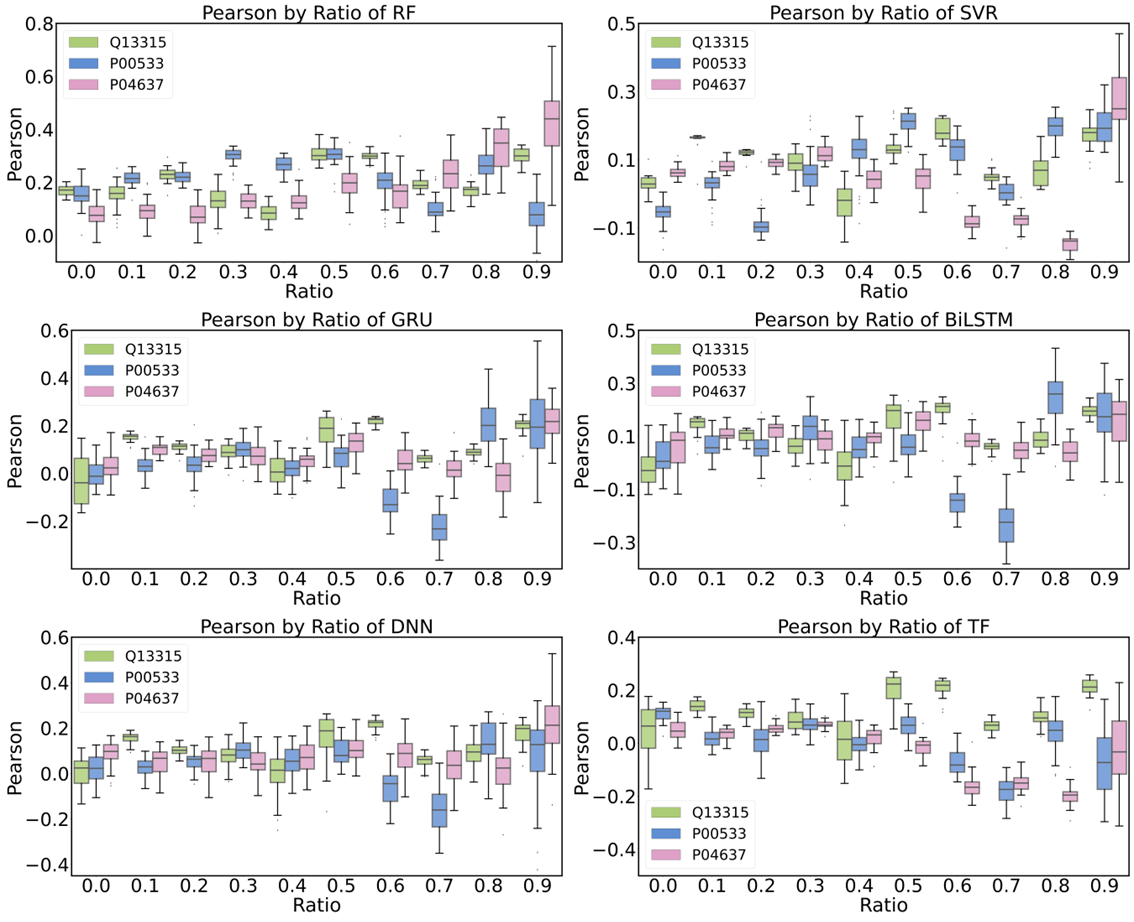


**Fig. S6** Pearson coefficient changes with simple gradual introduction of reference data for three systems using hyperparameters optimized based on UniProt-based partitioning.


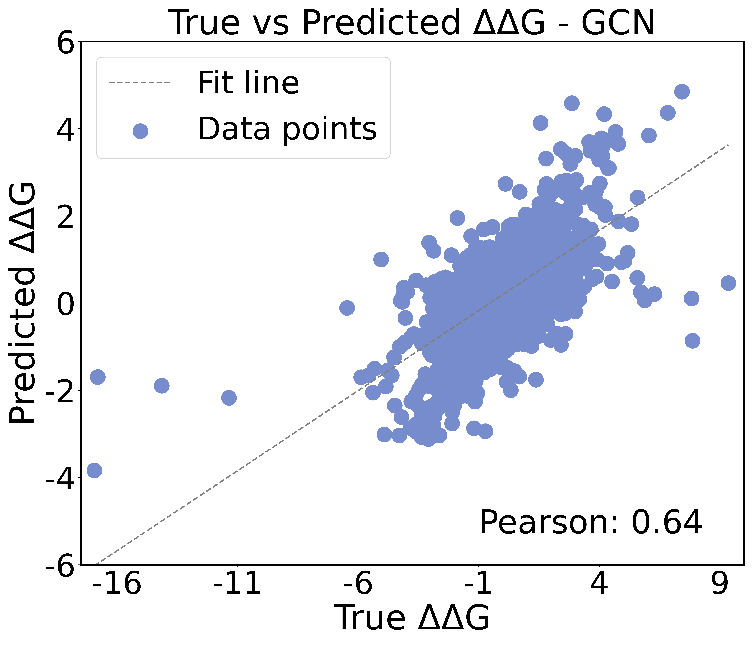


**Fig. S7**. True versus predicted relative binding free energy (ΔΔG) using a graph convolutional network (GCN). The unit of ΔΔG is kcal/mol.

Table S1. Performance evaluation of each model on the test set under random partitioning and UniProt-based partitioning. The best Pearson and Spearman coefficients are collected.

| **Model** | **MAE** | **RMSE** | **Best Pearson** | **Best Spearman** | **Partitioning** |
| --- | --- | --- | --- | --- | --- |
| RF | **0.76±0.04** | 1.15±0.12 | 0.66 | **0.61** | Random |
| RF | 0.99±0.14 | 1.48±0.34 | 0.24 | 0.29 | UniProt |
| SVR | 0.78±0.04 | 1.15±0.13 | 0.61 | 0.58 | Random |
| SVR | 1.31±0.17 | 1.75±0.28 | 0.32 | 0.36 | UniProt |
| GRU | 0.78±0.04 | 1.16±0.14 | 0.63 | 0.60 | Random |
| GRU | 1.05±0.13 | 1.46±0.26 | 0.24 | 0.27 | UniProt |
| BiLSTM | 0.81±0.04 | 1.22±0.15 | **0.70** | 0.58 | Random |
| BiLSTM | 1.02±0.15 | 1.40±0.31 | 0.27 | 0.33 | UniProt |
| DNN | 0.77±0.03 | **1.13±0.12** | 0.66 | 0.60 | Random |
| DNN | 1.01±0.12 | 1.41±0.27 | 0.25 | 0.37 | UniProt |
| Transformer | 0.78±0.03 | 1.16±0.09 | 0.62 | 0.59 | Random |
| Transformer | 1.09±0.17 | 1.52±0.28 | 0.23 | 0.27 | UniProt |
